# Characterisation of Naturally Occurring MERS-CoV Spike Mutations and Their Impact on Fusion and Neutralisation

**DOI:** 10.3390/v18030377

**Published:** 2026-03-18

**Authors:** Rachael Dempsey, Hannah Goldswain, Joseph Newman, Nazia Thakur, Tracy MacGill, Todd Myers, Robert Orr, Dalan Bailey, James P. Stewart, Waleed Aljabr, Julian A. Hiscox

**Affiliations:** 1 Institute of Infection, Veterinary and Ecological Sciences, Faculty of Health and Life Sciences, University of Liverpool, Liverpool L69 7ZB, UK; 2 The Pirbright Institute, Pirbright, Woking, Surrey GU24 0NF, UK; 3 Office of Regulatory and Emerging Science, U.S. Food and Drug Administration, Silver Spring, MD 20993, USA; 4 The Pandemic Institute, Liverpool L7 3FA, UK; 5 Public Health Authority, Riyadh 13351, Saudi Arabia; 6 King Fahad Medical City, Riyadh 12231, Saudi Arabia; 7 A*STAR Infectious Diseases (ID) Labs, Singapore 138648, Singapore

**Keywords:** MERS-CoV, Spike, Fusion, Pseudotypes, Neutralisation

## Abstract

In this study, the phenotypic consequences of naturally occurring single nucleotide polymorphisms (SNPs) in the Middle East respiratory syndrome coronavirus (MERS-CoV) Spike protein were investigated. The impact of Spike mutations on the syncytia formation and neutralisation of contemporary MERS-CoV strains is not currently well understood. Mutations were identified by aligning 584 MERS-CoV Spike sequences from either human clinical isolates collected between 2012 and 2024 or from a clinical isolate that had been passaged in human cells. Fifteen SNPs of interest occurring in the N-terminal domain (NTD), receptor binding domain (RBD) and adjacent to the S1/S2 cleavage site were selected for further characterisation based on their location in the Spike protein, frequency and identification in previous studies. A contemporary clade B, lineage 5 wildtype Spike sequence, obtained from a human MERS-CoV clinical isolate, was used as the backbone in this study. The mutations of interest were introduced to the wildtype backbone to generate Spike variants. Spike variants were characterised via cell–cell fusion assays, and a lentiviral pseudotyping system was used to investigate the impact of these Spike mutations on neutralisation. The I529T, E536K and L745F mutations were shown to increase fusion and syncytia formation. The L411F, T424I, L506F, L745F and T746K mutations were found to increase resistance to neutralisation by pooled patient sera. This study has identified novel naturally occurring Spike mutations that resulted in phenotypic differences in the syncytia formation and neutralisation of contemporary MERS-CoV strains. Continued investigation of the phenotypic consequences of MERS-CoV Spike mutations is essential for assessing the risk to public health, especially given the pandemic potential of this virus.

## 1. Importance

The main aim of this study was to investigate the impact of MERS-CoV Spike mutations on fusion and neutralisation. The phenotypic consequences of mutations occurring in the Spike protein of contemporary MERS-CoV strains during human infections are not currently well understood. Improving our understanding is of particular importance given that MERS-CoV continues to pose a public health risk, with frequent spillover events and mounting evidence of human-to-human transmission since the virus emerged in 2012. A major concern is that as MERS-CoV continues to evolve, it may become more infectious, resulting in increased transmission between humans. To add to this, surveillance is limited and there are currently no specific medical countermeasures available to treat MERS-CoV disease. The MERS-CoV Spike pseudotyping system developed in this study is a useful tool that could be used alongside surveillance systems to rapidly assess novel Spike mutations in functional assays. This MERS-CoV pseudotyping system could also be used to aid in the development of medical countermeasures such as vaccines, antivirals and antibody therapies.

## 2. Introduction

MERS-CoV evolves via recombination, single nucleotide polymorphisms (SNPs), insertions and deletions (indels) [1]. Since emerging in 2012, three genetically distinct clades have been identified; clades A, B and C [2,3,4]. The MERS-CoV Erasmus Medical Centre/2012 (EMC/2012) reference strain, isolated from the first patient identified as being infected with the virus in 2012, was phylogenetically classified as clade A and has been the focus of most MERS-CoV research studies to date. However, clade A viruses were outcompeted by clade B viruses and have not been detected since 2015 [4,5]. Clade B viruses continue to circulate in dromedary camels and humans, predominantly in the Middle East [4,6], and have been the cause of epidemic outbreaks of MERS-CoV since 2012 [7]. The most notable outbreaks in humans occurred in Saudi Arabia in 2014 and South Korea in 2015 [8,9], where the index case was a traveller returning from the Middle East [9,10,11]. Human-to-human transmission was observed during these outbreaks [9,10,11,12], which were associated with a recombination event between clade B viruses belonging to lineages 3 and 4 [13]. The recombination event occurred at ORF1ab/Spike and resulted in a new lineage: clade B lineage 5 (B5). B5 viruses were of superior replicative fitness and caused lower levels of cytokine and interferon (IFN) induction relative to lineages 3 and 4, which may have resulted in the increased number of human cases during these outbreaks [7,13]. Clade C viruses are predominantly present in camels in African countries, with minimal human infections observed for this clade [2,3,14].

Clade B viruses have been phylogenetically classified into seven lineages, indicating the genome diversity and continued evolution of the viruses in this clade [4]. SNPs occurring in Spike are of particular interest because this protein mediates MERS-CoV entry into host cells. Spike is also the main target of neutralising antibodies [15], and SNPs in the gene that encodes for this protein may lead to partial immune escape in individuals that have previously experienced a natural MERS-CoV infection. Additionally, Spike is a target for both human and camel vaccines being trialled to protect against MERS-CoV infection [16,17,18,19,20,21]. Therefore, changes in Spike may confer an advantage to the virus by improving entry and making MERS-CoV more infectious [22], or by leading to partial immune escape and reducing immunity in individuals previously infected with MERS-CoV [23]. Improved entry and/or partial immune escape of MERS-CoV could result in increased virus transmission in humans, which is a major public health concern.

The Spike protein exists as a trimer at the surface of the viral envelope, with each monomer carrying a non-covalently associated S1 and S2 subunit after cleavage at S1/S2 [24,25,26]. The S1 subunit contains the N-terminal domain (NTD) responsible for attachment to the host cell [27,28,29] and the receptor-binding domain (RBD) that interacts with the dipeptidyl peptidase-4 (DPP4) receptor [30,31]. The S2 subunit mediates membrane fusion and contains the S2′ cleavage site, fusion peptide, heptad repeat domains 1 and 2, transmembrane domain and cytoplasmic domain [26]. Spike binds the DPP4 receptor on the host cell surface via its S1 subunit, which subsequently leads to cleavage at S2′ [26,30]. This cleavage event enables the fusion of viral and host membranes via the S2 subunit and entry of the virus into the host cell [26]. Entry into the host cell can be prevented by neutralising antibodies binding to the virus. The majority of MERS-CoV neutralising antibodies bind to the S1 subunit, usually to the RBD, although NTD-specific neutralising antibodies have also been identified [15]. Neutralising antibodies that bind to the S2 subunit are less common and tend to be of lower potency than those that bind to Spike S1 [32,33].

The main aim of this study was to investigate the phenotypic impact of Spike mutations in contemporary MERS-CoV strains to improve our understanding of human infections. Mutations were identified by aligning MERS-CoV Spike sequences from human clinical isolates collected between 2012 and 2024 or from viruses that had been passaged in human cells. Fifteen mutations of interest were selected for further characterisation based on their location within the Spike protein, mutation frequency and inclusion in previous studies. The mutations selected included SNPs located in the NTD, RBD and adjacent to the S1/S2 cleavage site. Mutations were introduced into a contemporary clade B, lineage 5 wildtype Spike backbone, obtained from a clinical isolate collected in Saudi Arabia in 2019. This backbone was selected because it was the most recent human isolate available when this study commenced, and it belongs to the B5 clade, which continues to circulate in camels and humans in the Middle East. Spike mutations were then characterised via cell–cell fusion assays, and a lentiviral pseudotyping system was used to investigate the impact of these Spike mutations on neutralisation [34,35].

Improving our understanding of genotype-to-phenotype changes in MERS-CoV Spike is of particular importance owing to the pandemic potential of this virus. Since emerging in 2012, frequent spillover events have occurred and several instances of human-to-human transmission have been identified [36,37]. The most recent examples were nine cases of MERS-CoV reported to the World Health Organisation (WHO) in April 2025, with seven cases originating from one index case [38]. Additionally, in early December 2025, two MERS-CoV cases were reported in travellers returning to France from the Arabian Peninsula [39]. This highlights the importance of continued investigation of contemporary MERS-CoV strains, as with each spillover event the virus continues to evolve and adapt [37], which may lead to increased infectivity and transmission in humans.

## 3. Methods

### 3.1. MERS-CoV Spike Sequence Alignment

MERS-CoV Spike sequences used in this study were obtained from GenBank (NCBI) (n = 579), the Saudi Arabian Public Health Authority (n = 1) and from our own in-house sequencing of reverse genetics viruses passaged in human cells (n = 4) using an amplicon-based sequencing approach that has previously been described [40]. The 580 human isolates in this alignment were collected between 2012 and 2024 and predominantly included isolates from Saudi Arabia, although some isolates collected in the UAE, Jordan, Qatar and South Korea were also included. The remaining four sequences that were included in the alignment were obtained after passaging a clade B, lineage 5 clinical isolate (Saudi Arabia, 2019) four times in human cells. In total, 584 Spike sequences were aligned using the Multiple Sequence Alignment with MUSCLE tool in Unipro UGENE software (Unipro LLC, Novosibirsk, Russia). The Spike gene from the 2019 clinical isolate collected in Saudi Arabia was used as the reference sequence in this alignment (Supplementary Figure S1). Spike S1 amino acid changes relative to the 2019 clinical isolate and their frequency were identified and recorded. Fifteen SNPs of interest occurring in the NTD, RBD and adjacent to the S1/S2 cleavage site were selected for further characterisation. Mutations of interest were selected based on their frequency and locations within the Spike protein, as well as according to prior knowledge available from previous studies in the literature.

### 3.2. Cell Maintenance

Human embryonic kidney 293T (HEK-293T) cells and baby hamster kidney 21 (BHK-21) cells were purchased from the American Type Culture Collection (ATCC). Cells were cultured and maintained in Dulbecco’s modified Eagle’s medium—high glucose (DMEM; Sigma-Aldrich, Merck, Darmstadt, Germany) supplemented with 10% fetal bovine serum (FBS; Sigma-Aldrich), termed complete DMEM, at 37 °C with 5% CO_2_. Cells were passaged every 3–4 days.

### 3.3. Plasmids

Codon-optimised MERS-CoV Spike contained in the pcDNA3.1(+) expression vector was designed and synthesised using GeneArt™ (Thermo Fisher Scientific, Waltham, MA, USA). The Spike sequence was taken from a clinical isolate collected in Saudi Arabia in 2019 and belonged to the same clade as MERS-CoV viruses that were circulating in the Middle East (Supplementary Figure S1). The Kozak sequence was included immediately upstream and a C-terminal FLAG-tag was included downstream of the Spike sequence. The final 16 amino acids, containing an endoplasmic reticulum (ER)/Golgi retention motif and an endosomal recycling motif, were removed from the C-terminal cytoplasmic tail of Spike to increase pseudotyping efficiency [41]. This plasmid is referred to as the wildtype Spike plasmid. A no glycoprotein (GP) pcDNA3.1(+) vector containing a FLAG-tag (Addgene, Watertown, MA, USA) was used as an empty vector control, and VSV-G contained within the pcDNA3.1(+) vector was used as the positive control, as it is known to pseudotype efficiently. The lentiviral pseudotyping system consisted of two plasmids: p8.91 and pCSFLW. p8.91 is a second-generation packaging construct containing the HIV-1 core genes, *gag-pol*, under the control of a human CMV promoter [42]. The pCSFLW transfer plasmid contained the firefly luciferase reporter flanked by HIV-1 regulatory long terminal repeats (LTRs) and a packaging signal [43]. GFP1-7 and GFP8-11 were contained within the pcDNA3.1(+) expression vector and were used in the split GFP cell–cell fusion assays. Human DPP4 in a pCMV3-untagged expression vector was purchased from Sino Biological.

### 3.4. Plasmid Propagation

Plasmids were propagated by transforming into NEB^®^ 5-alpha competent *Escherichia coli* (*E. coli*) (high efficiency) cells (New England Biolabs; NEB, Ipswich, MA, USA) via heat shock, as detailed in the manufacturer’s instructions. Bacteria were then spread onto appropriate selective LB agar plates and incubated overnight at 37 °C to allow *E. coli* colony formation. Single colonies were picked from the LB agar plates and expanded in 250 mL selective LB media at 37 °C and 250 rpm overnight. Plasmid DNA was then purified from the *E. coli* cultures using the QIAGEN plasmid maxi kit (Qiagen, Hilden, Germany), as per manufacturer’s instructions. Plasmids were quantified using a Nanodrop One spectrophotometer (Thermo Scientific, Thermo Fisher Scientific) and verified via restriction endonuclease digest and Sanger sequencing (Eurofins Genomics, Ebersberg, Germany) prior to use in this study.

### 3.5. Site-Directed Mutagenesis

The plasmid containing the wildtype Spike sequence was used as a template for site-directed mutagenesis to produce plasmid variants carrying the mutations of interest. SNPs were introduced using the QuikChange Lightning site-directed mutagenesis kit (Agilent, Santa Clara, CA, USA), as detailed in the manufacturer’s instructions. The wildtype Spike plasmid (50 ng) was used as the template in each reaction, along with the appropriate mutagenesis primer pair for each mutation (Supplementary Table S1). Mutagenesis reactions were digested with 2 µL of DpnI, and 2 µL of each reaction was transformed into *E. coli* for plasmid propagation, as described above.

### 3.6. Transfection

Transfections were performed using Lipofectamine 3000 (Thermo Fisher), as detailed in the manufacturer’s instructions. Plasmids were transfected into HEK-293T cells to produce pseudotypes or for the split GFP cell–cell fusion assay, while plasmids were transfected into BHK-21 cells for the split GFP cell–cell fusion assay only. HEK-293T cells were seeded into 6-well tissue culture-treated plates (CytoOne, Starlab, Hamburg, Germany) coated with poly-L lysine (Sigma-Aldrich), as per the manufacturer’s instructions. BHK-21 cells were seeded into 6-well tissue culture-treated plates (CytoOne, Starlab). Cells were seeded at an appropriate density 24 h prior to transfection to produce cell monolayers that were 70–80% confluent at the point of transfection. Transfections were performed in Opti-MEM™ media (Thermo Fisher) using 7.5 µL of Lipofectamine 3000, 5 µL of P3000 reagent and the appropriate plasmid concentrations, as described previously [34,44,45]. After incubating for 15 min at room temperature, transfection mixes were added to the cells and incubated overnight at 37 °C with 5% CO_2_. At 18 h post-transfection, transfection mixes were removed and replaced with 3 mL of complete DMEM. Cell plates were then incubated at 37 °C with 5% CO_2_ for a further 24–48 h.

### 3.7. Generation of Pseudotypes

Pseudotypes were generated via co-transfection of the p8.91 packaging plasmid, the pCSFLW transfer plasmid and the different variants of the MERS-CoV Spike plasmid using Lipofectamine 3000 (Thermo Fisher), as described above. The plasmid concentrations used in each transfection reaction were 0.6 µg of p8.91, 0.6 µg of pCSFLW and 0.5 µg of Spike, as previously described [34,35]. Pseudotypes were harvested at 48 and 72 h post-transfection and pooled. Pseudotypes were centrifuged at 300 g for 10 min at 4 °C, before being aliquoted and stored at −80 °C. Pseudotype cell lysates were also collected for use in western blot analyses by adding 250 µL of Laemmli buffer (Sigma-Aldrich) combined with 250 µL of PBS (Sigma-Aldrich) to each well and pipetting up and down to disrupt the cell monolayer.

### 3.8. Titration Assays

Titration and viability of pseudotypes were tested using a luciferase-based infectivity assay, as previously described [34]. Four days prior to pseudotype infection, HEK-293T cells were seeded at an appropriate density into 6-well tissue culture-treated plates (CytoOne, Starlab) coated with poly-L lysine (Sigma-Aldrich), as described above. After 24 h, HEK-293T cells were transfected with 0.5 µg of human DPP4 using Lipofectamine 3000 (Thermo Fisher), as described above. At 18 h post-transfection, the media was replaced with complete phenol red-free DMEM and cell plates were incubated for a further 24 h to allow for DPP4 expression.

On the day prior to pseudotype infection, HEK-293T DPP4 cells were washed and collected by pipetting complete phenol red-free DMEM up and down to disrupt the cell monolayer. Cells were counted and seeded into 96-well white tissue culture-treated plates (Sigma-Aldrich) at a density of 2 × 10^4^ cells per well in complete phenol red-free DMEM and incubated at 37 °C with 5% CO_2_ for 24 h. On the day of pseudotype infection, all pseudotypes were serially diluted 10-fold from neat to 1 × 10^−4^ in phenol red-free DMEM—high glucose (Sigma-Aldrich) supplemented with 2% FBS (Sigma-Aldrich). Next, 100 µL of the serially diluted pseudotypes was added to each well and the plates were incubated at 37 °C with 5% CO_2_ for 72 h. After 72 h, the media was removed and 50 µL of Bright-Glo™ (Promega, Madison, WI, USA) substrate combined 1:1 with phenol red-free DMEM was added per well and incubated in the dark for five minutes at room temperature. Luciferase activity was then measured using a GloMax^®^ Discover microplate reader (Promega) with a 0.5 s integration time.

### 3.9. Split GFP Cell–Cell Fusion Assays

Split GFP cell–cell fusion assays were used to investigate syncytia formation with the Spike proteins carrying different mutations and were performed as previously described [34,45]. Three days prior to the split GFP cell–cell fusion assay, HEK-293T and BHK-21 cells were seeded at appropriate densities in 6-well tissue culture-treated plates (CytoOne, Starlab), as described above. After 24 h, HEK-293T cells were co-transfected with 0.25 µg of Spike and 0.5 µg of GFP1-7, and BHK-21 cells were co-transfected with 0.5 µg of human DPP4 and 0.5 µg of GFP8-11 using Lipofectamine 3000 (Thermo Fisher), as described above. After replacing the media with complete phenol red-free DMEM 18 h post-transfection, the cell plates were incubated for a further 24 h to allow for protein expression.

Split GFP cell–cell fusion assays were then performed by co-culturing HEK-293T cells expressing Spike and GFP1-7 with BHK-21 cells expressing the DPP4 receptor and GFP8-11. On the day of co-culturing, the media was removed, and cells were washed and collected by pipetting phenol red-free DMEM (Sigma-Aldrich) up and down to disrupt the cell monolayer. Cells were counted and seeded into a 96-well plate (CytoOne, Starlab) at a density of 5 × 10^4^ cells/well in 100 µL of phenol red-free DMEM—high glucose (Sigma-Aldrich) supplemented with 2% FBS (Sigma-Aldrich). Cells were incubated at 37 °C with 5% CO_2_ for 1 h to allow cells to settle and adhere to the plate. After 1 h, cell plates were transferred to an IncuCyte^®^ (Sartorius, Göttingen, Germany), where they were incubated at 37 °C with 5% CO_2_ and GFP expression and syncytia formation were measured from images taken every 2 h for 24 h.

### 3.10. Microneutralisation Assays

Microneutralisation assays were used to investigate whether the Spike mutations impacted neutralisation by pooled patient sera and were performed as previously described [34,44]. The pooled patient sera used in these assays was the 1st WHO International Standard for Anti-MERS-CoV Immunoglobulin G (Human) 19/178 purchased from the National Institute for Biological Standards and Control (NIBSC). It contained the pooled serum from two individuals from South Korea who had recovered from MERS-CoV infection [46].

Three days prior to pseudotype infection, HEK-293T cells were seeded at an appropriate density into 6-well tissue culture-treated plates (CytoOne, Starlab) coated with poly-L lysine (Sigma-Aldrich), as described above. After 24 h, HEK-293T cells were transfected with 0.5 µg of human DPP4 using Lipofectamine 3000 (Thermo Fisher), as described above. At 18 h post-transfection, the media was replaced with complete phenol red-free DMEM, and cell plates were incubated for a further 24 h to allow for DPP4 expression. The pooled patient sera was then diluted 1:10 in phenol red-free DMEM—high glucose (Sigma-Aldrich) supplemented with 2% FBS (Sigma-Aldrich) and serially diluted 2-fold to 1:2560 in 96-well white tissue culture-treated plates (Sigma-Aldrich). Pseudotypes at a titre equivalent to 10^5^–10^6^ relative light units (RLU) were then combined 1:1 with the pooled patient sera and incubated for 1 h at 37 °C with 5% CO_2_. During this incubation, HEK-293T DPP4 cells were washed and collected in phenol red-free DMEM—high glucose (Sigma-Aldrich) supplemented with 2% FBS (Sigma-Aldrich) by pipetting up and down. After incubating the pseudotypes and sera for 1 h, cells were seeded at a density of 2 × 10^4^ cells per well in the 96-well white plates containing the pseudotypes and sera. The plates were then incubated for 72 h at 37 °C with 5% CO_2_. After 72 h, the media was removed and replaced with 50 µL of Bright-Glo™ (Promega) substrate combined 1:1 with phenol red-free DMEM—high glucose (Sigma-Aldrich). Plates were incubated in the dark for five minutes at room temperature prior to luciferase activity being measured using a GloMax^®^ Discover microplate reader (Promega) with a 0.5 s integration time.

### 3.11. Statistical Analysis

Data were tested for normal and log-normal distributions to dictate the statistical tests used. Subsequent analyses are described in the figure legends. All graphs were generated in GraphPad Prism, version 10. Statistical analyses were performed in GraphPad Prism, version 10.

## 4. Results

### 4.1. Identification of Mutations of Interest in MERS-CoV Spike Protein

Naturally occurring MERS-CoV Spike mutations identified in human infections may result in phenotypic differences in virus entry and neutralisation. Improved virus entry or partial immune escape could pose a significant risk to human health. To investigate this, amino acid mutations were identified by aligning the Spike genes from 584 human MERS-CoV sequences. A clade B, lineage 5 virus collected in Saudi Arabia in 2019 was used as the wildtype Spike sequence backbone (Supplementary Figure S1). This clinical isolate sequence was selected as it belongs to the same clade as the viruses currently circulating in the Middle East, as well as being the most recently available human MERS-CoV isolate when this study commenced. This 2019 clinical isolate contains five amino acid changes in its Spike sequence relative to the EMC/2012 reference strain (Table 1). The Q1020R amino acid change identified in the 2019 clinical isolate was also carried by all 584 human MERS-CoV sequences included in the alignment. The four remaining amino acid changes present in the 2019 clinical isolate when compared to EMC/2012 were P387T, L717I, R1179M and E1183D. P387T was present in 6 sequences, L717I was present in 5 sequences, R1179M was present in 12 sequences and E1183D was present in 4 of the 584 human MERS-CoV sequences in the alignment.

**Figure 1:**
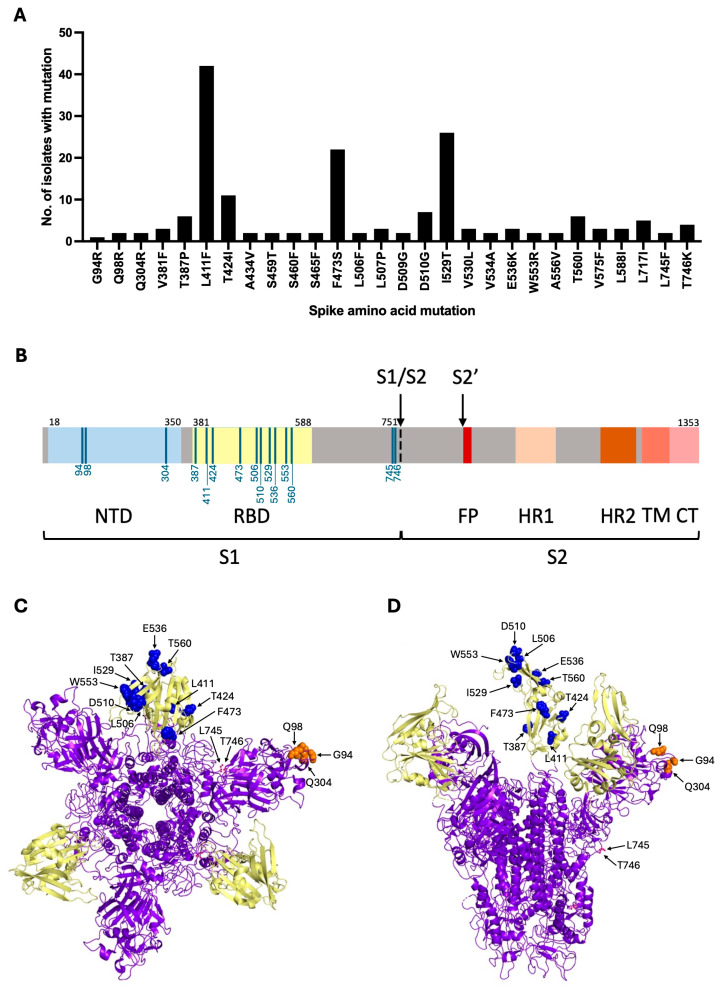
Summary of Spike S1 mutations identified in an alignment of 584 MERS-CoV clinical isolates. (**A**) Mutations that were identified are shown on the x-axis and have been plotted against the number of clinical isolates carrying the mutation. (**B**) Schematic showing the different domains of Spike with the locations of the mutations that were selected for further characterisation labelled. Mutations were situated in the N-terminal domain (NTD; blue), receptor binding domain (RBD; yellow) and adjacent to the S1/S2 cleavage site (black arrow). The S2′ cleavage site (S2′; black arrow), fusion peptide (FP; red), heptad repeat region 1 (HR1; peach), heptad repeat region 2 (HR2; brown), transmembrane domain (TM; coral) and cytoplasmic tail (CT; pink) are also depicted. (**C**,**D**) Top and side views of the Spike protein structure in a pre-fusion state (Protein databank structure: 5X59; https://doi.org/10.2210/pdb5X59/pdb) with the locations of the mutations of interest highlighted. The RBD is shown in yellow with the locations of RBD mutations highlighted in blue. Locations of NTD mutations are highlighted in orange and cleavage site mutations are in pink.

Twenty-eight SNPs were identified in Spike S1 in this alignment, and their frequencies were recorded (Figure 1A). G94R, Q98R and Q304R were identified when the 2019 clinical isolate was passaged in human cells, while the remaining SNPs were naturally occurring mutations identified in the human MERS-CoV isolates. The majority of the mutations identified were located in the RBD [24] (amino acids 381–588; Figure 1A–D). G94R, Q98R and Q304R were located in the NTD, while L745F and L746K were located adjacent to the S1/S2 cleavage site. L411F, F473S and I529T were the most commonly occurring mutations, with 42, 22 and 26 isolates containing each of these mutations, respectively (Figure 1A). Mutations of interest were then selected for further characterisation based on location, frequency and the likelihood of impacting entry and/or the neutralisation properties of the virus. The following 15 mutations were down-selected: G94R, Q98R, Q304R, T387P, L411F, T424I, F473S, L506F, D510G, I529T, E536K, W553R, T560I, L745F and T746K (Figure 1; Table 2). These mutations were found independently in separate isolations of the virus and never together. Therefore, in this study, mutations were characterised as single point mutations only.

The locations of the 15 mutations of interest were mapped to show their positions relative to the different Spike domains and locations within the Spike protein structure (Figure 1B–D). Mutations located in the RBD were likely to directly interact with the DPP4 receptor and therefore impact entry. Additionally, many of the RBD residues were exposed on the outer surface of the Spike S1 subunit (Figure 1C,D). This suggested that the RBD residues could be targeted by neutralising antibodies and mutations occurring in these positions may therefore impact neutralisation. Mutations located in the NTD were of interest due to their possible role in host cell attachment and their exposed locations on the outer surface of the Spike S1 subunit (Figure 1C,D) [27,28,29], making them possible targets for neutralising antibodies. Mutations located close to the S1/S2 cleavage site were selected on the basis that they may alter cleavage efficiency and therefore impact entry. L745 and T746 were also located on the outer surface of the Spike protein (Figure 1D) and may therefore impact neutralisation if this region is targeted by antibodies.

### 4.2. Generation of MERS-CoV Spike Lentiviral Pseudotypes

To evaluate the effect of different mutations on neutralisation properties, MERS-CoV Spike lentiviral pseudotypes were generated through co-transfection of the lentiviral pseudotyping system and Spike expression plasmid into HEK-293T cells. This resulted in Spike being incorporated into nascent lentiviral particles to produce replication-deficient pseudotypes. Pseudotypes were then subsequently titrated prior to being used to study the neutralisation properties of Spike proteins carrying the different mutations of interest.

The plasmids used to generate pseudotypes in this study were the p8.91 packaging plasmid, pCSFLW transfer plasmid and plasmids containing the Spike variants. Spike variant plasmids containing the mutations of interest were generated by site-directed mutagenesis of the wildtype Spike plasmid. VSV-G was included as a positive control for pseudotype generation and titration as it is known to pseudotype efficiently and no GP empty pcDNA3.1(+) vector (Addgene) was included as a negative control. Plasmids were verified by restriction endonuclease digest and submitted to Eurofins Genomics for Sanger sequencing, which confirmed that all Spike plasmid variants contained the expected mutations. The lentiviral pseudotyping system and Spike plasmids were then used to generate pseudotypes via co-transfection into HEK-293T cells followed by harvesting of cell supernatants containing the pseudotypes after 48 h and 72 h.

After the pseudotypes were harvested, cell lysates were collected for analysis via western blot to confirm the expression of Spike, the HIV-1 lentiviral *gag* and *pol* core genes, the firefly luciferase reporter and VSV-G. Western blot analysis indicated that protein expression was as expected for each of the pseudotype cell lysates (Supplementary Figures S2–S4). Spike expression was confirmed in all pseudotype cell lysates, except for the VSV-G and no GP controls. Expression of VSV-G was only observed in the VSV-G pseudotype cell lysate. Expression of the HIV-1 core and the firefly luciferase reporter was observed for all pseudotypes, including the VSV-G and no GP controls. GAPDH was included as a loading control and was detected across all samples, including the no plasmid transfection control, as expected. Spike, the HIV-1 lentiviral core and the firefly luciferase reporter were not found to be expressed in the no plasmid transfection control cell lysate. This indicated that protein expression was as expected for all pseudotypes and controls.

### 4.3. Titres of Pseudotypes Carrying Mutations in the Spike NTD Were Reduced Relative to That of the Wildtype

**Figure 2:**
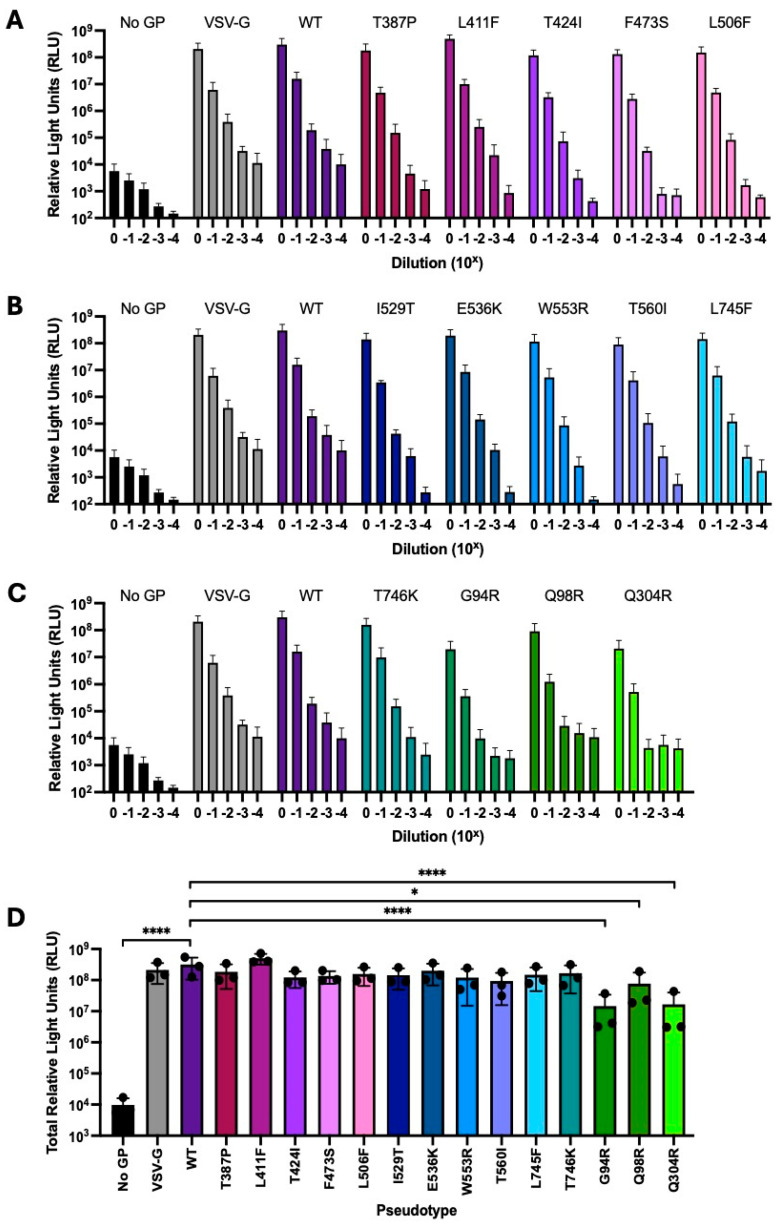
Titres of pseudotypes carrying NTD mutations were reduced relative to that of the wildtype. Pseudotypes were serially diluted 10-fold and used to infect HEK-293T cells expressing DPP4. (**A**–**C**) Luciferase activity was measured 72 h post-infection using a GloMax^®^ Discover microplate reader (Promega). (**D**) Total luciferase activity across the dilution series was plotted to include all dilutions simultaneously in the statistical analysis. One-way ANOVA with Dunnet’s multiple comparisons test indicated that titres of no GP, G94R, Q98R and Q304R pseudotypes were significantly reduced relative to that of the wildtype (n = 3 independent replicates; error bars show mean with standard deviation; * *p* < 0.05, **** *p* < 0.0001).

Once correct protein expression had been confirmed, the pseudotypes were titrated in a luciferase-based infectivity assay to verify that they were functional. Functional pseudotypes should be able to enter HEK-293T DPP4 cells via binding to the DPP4 receptor, which will subsequently result in expression of the firefly luciferase reporter. Spike wildtype, Spike mutants 1–15, VSV-G and no GP pseudotypes were serially diluted 10-fold and used to infect HEK-293T DPP4 cells. After 72 h, luminescence was measured to determine pseudotype titres, with the no GP negative control being indicative of background (Figure 2A–C). Undiluted pseudotypes with titres >2.0 Log_10_ RLU above the no GP negative control were considered to be infective and therefore functional (Figure 2A–C) [34].

Pseudotype titres were then expressed as total RLU to generate a single data point per pseudotype in each experiment for statistical analysis, while still using all data points across the dilution series (Figure 2D). Total RLU titres for mutants 1–15 and the positive and negative controls were compared to that of the wildtype Spike pseudotype. Pseudotypes carrying the NTD mutations G94R, Q98R or Q304R were found to have significantly reduced titres relative to the wildtype (*p* < 0.0001, *p* < 0.05 and *p* < 0.0001, respectively). The titres of the remaining pseudotypes (mutants 1–12) were comparable to that of the wildtype. The titre of the no GP negative control was significantly reduced relative to that of the wildtype Spike pseudotype (*p* < 0.0001), as expected.

### 4.4. I529T, E536K and L745F Mutations Increased Fusion Relative to the Wildtype

Split GFP cell–cell fusion assays were performed to assess the impact of the Spike mutations on receptor binding and membrane fusion. The NTD has been shown to play a role in host cell attachment [27,28,29], meaning that mutations located in the NTD (G94R, Q98R and Q304R) may impact entry. Mutations located in the RBD (T387P, L411F, T424I, F473S, L506F, D510G, I529T, E536K, W553R and T560I) may alter receptor-binding properties. Mutations located near the S1/S2 cleavage site (L745F and T746K) may impact cleavage efficiency, which would have a knock-on effect on virus entry.

**Figure 3:**
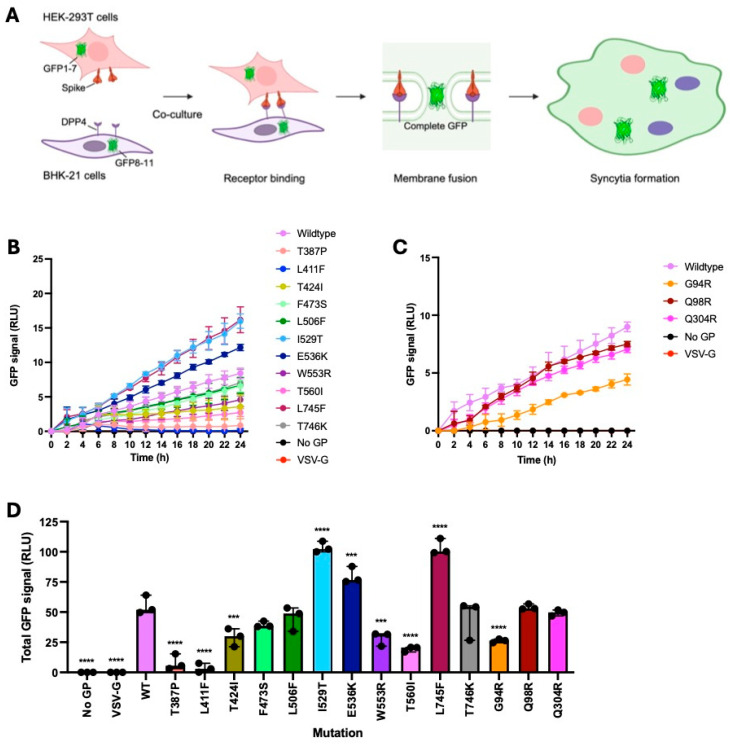
Receptor binding and membrane fusion of I529T, E536K and L745F mutants were improved relative to the wildtype. (**A**) Schematic depicting the formation of syncytia during the split GFP cell–cell fusion assay. HEK-293T cells expressing Spike and GFP1-7 were co-cultured with BHK-21 cells expressing DPP4 and GFP8-11. Spike binds the DPP4 receptor, leading to membrane fusion and syncytia formation, resulting in the two halves of GFP being assembled into a functional fluorescent protein. (**B**,**C**) GFP signal emitted by the Spike mutants and controls over the 24 h period following co-culturing, normalised to background controls. The GFP signal was measured every 2 h using an IncuCyte^®^ (Sartorius). (**D**) Total GFP signal across the time course was plotted to include all time points simultaneously in the statistical analysis. One-way ANOVA with Dunnet’s multiple comparisons test indicated that the I529T, E536K and L745F mutations significantly improved receptor binding and membrane fusion relative to the wildtype. The T387P, L411F, T424I, W553R, T560I and G94R mutations significantly reduced receptor binding and membrane fusion relative to the wildtype (n = 3 independent replicates; error bars show mean with standard deviation; *** *p* < 0.001, **** *p* < 0.0001).

To set up the split GFP cell–cell fusion assays, HEK-293T cells expressing wildtype Spike or a Spike variant and GFP1-7 were co-cultured with BHK-21 cells expressing the DPP4 receptor and GFP8-11 (Figure 3A). Spike binds to the DPP4 receptor, subsequently resulting in membrane fusion and syncytia formation. The two halves of GFP can then be assembled into a functional protein, emitting a fluorescent signal corresponding to the receptor binding and membrane fusion ability of the Spike protein. GFP expression was measured from images taken of the co-cultured cells every 2 h for 24 h using an IncuCyte^®^ (Sartorius) (Figure 3B,C). HEK-293T cells expressing VSV-G (glycoprotein negative control) and no GP pcDNA3.1(+) (no glycoprotein negative control) were also included in this experiment. Background fluorescence measurements taken from HEK293T cells expressing Spike and GFP1-7 only and BHK-21 cells expressing DPP4 and GFP8-11 only (not co-cultured) were averaged and subtracted from the values for the co-cultured conditions.

Fluorescence readings were then expressed as total RLU to generate a single data point per Spike protein in each experiment for statistical analysis, while still using all data points from the time course (Figure 3D). Total RLU titres for mutants 1–15 and the controls were compared to that of the wildtype Spike protein. Fusion was significantly increased for the Spike proteins carrying the I529T (*p* < 0.0001), E536K (*p* < 0.001) and L745F (*p* < 0.0001) mutations compared to that of the wildtype. By contrast, fusion was significantly reduced for the Spike proteins carrying the T387P (*p* < 0.0001), L411F (*p* < 0.0001), T424I (*p* < 0.001), W553R (*p* < 0.001), T560I (*p* < 0.0001) and G94R (*p* < 0.0001) mutations relative to that of the wildtype. Fusion of the Spike proteins carrying the F473S, L506F, T746K, Q98R and Q304R mutations was comparable to that of the wildtype. No receptor binding and membrane fusion was observed for the VSV-G and no GP negative controls, as expected.

**Figure 4:**
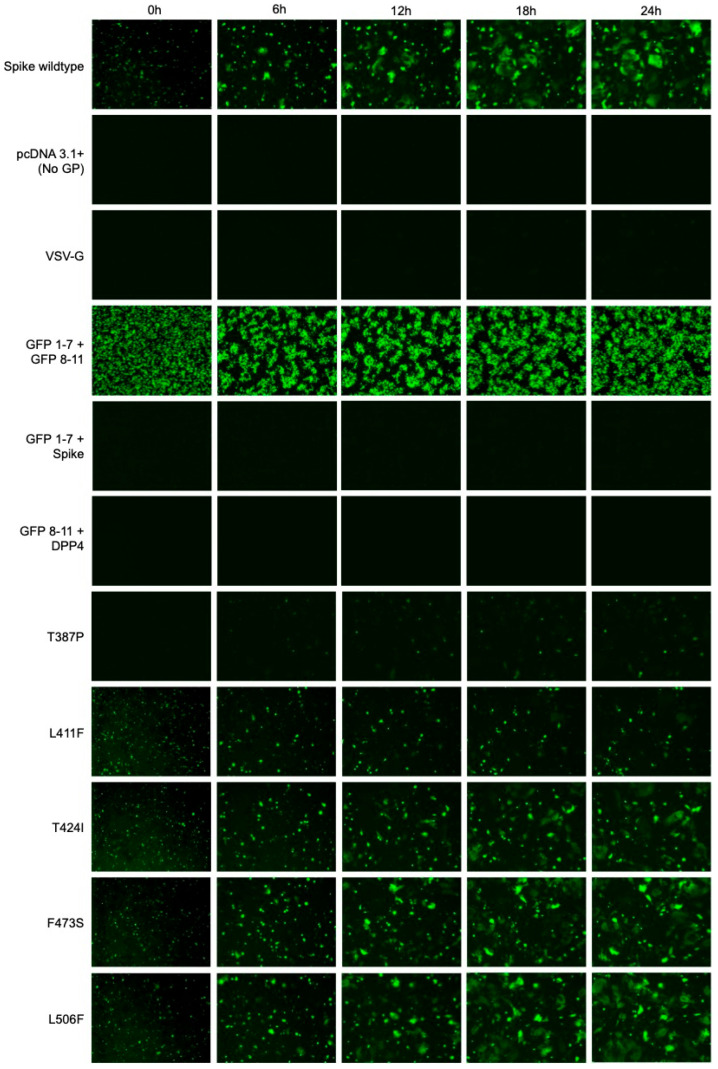
T387P, L411F and T424I mutants demonstrated decreased syncytia formation compared to the wildtype. HEK-293T cells expressing GFP1-7 and either wildtype Spike, Spike mutants 1–5, pcDNA3.1(+) (no GP) or VSV-G were co-cultured with BHK-21 cells expressing the DPP4 receptor and GFP8-11. GFP1-7 + GFP8-11, GFP1-7 + Spike and GFP8-11 + DPP4 were included as controls and to measure the background signal for normalisation. The GFP signal was measured from images taken every 2 h on the IncuCyte^®^ (Sartorius). Representative images taken at 0 h, 6 h, 12 h, 18 h and 24 h post-co-culture, with the GFP signal normalised to the whole plate, are shown here.

**Figure 5:**
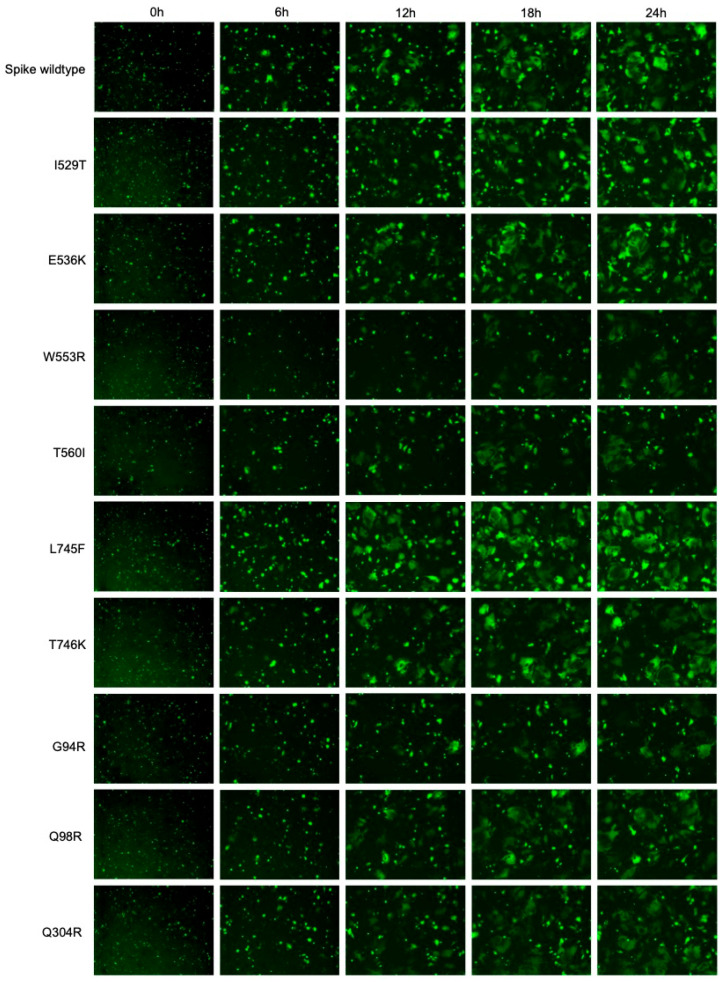
I529T, E536K and L745F mutants demonstrated increased syncytia formation compared to the wildtype. HEK-293T cells expressing GFP1-7 and either wildtype Spike or Spike mutants 7–15 were co-cultured with BHK-21 cells expressing the DPP4 receptor and GFP8-11. The GFP signal was measured from images taken every 2 h on the IncuCyte^®^ (Sartorius). Representative images taken at 0 h, 6 h, 12 h, 18 h and 24 h post-co-culture, with the GFP signal normalised to the whole plate, are shown here.

In addition to quantifying fusion by obtaining fluorescence readings from the images taken using the IncuCyte^®^ (Sartorius), syncytia were also visualised using these images and normalised to the whole plate. Representative fluorescence images taken at 0 h, 6 h, 12 h, 18 h and 24 h are shown for each experimental condition (Figure 4 and Figure 5). Syncytia formation for each Spike protein increased over time, indicating that receptor binding and membrane fusion increased over the 24 h experiment. Spike proteins carrying the I529T, E536K and L745F mutations demonstrated increased syncytia formation and GFP expression relative to the wildtype (Figure 5). Spike proteins carrying the T387P, L411F, T424I, W553R, T560I and G94R mutations demonstrated decreased syncytia formation and GFP expression relative to the wildtype (Figure 4 and Figure 5).

Importantly, no syncytia formation or GFP expression was observed for the VSV-G and pcDNA3.1(+) no GP controls (Figure 4). The GFP1-7 + Spike only and GFP8-11 + DPP4 only controls were included for background fluorescent measurements and no syncytia formation or GFP expression were observed in the images for these conditions. As expected, GFP expression but not syncytia formation was observed for GFP1-7 + GFP8-11 co-transfected into HEK-293T cells as a GFP expression control (Figure 4).

### 4.5. Pseudotypes Carrying the L411F, T424I, L506F, L745F and T746K Mutations Were Neutralised Less Efficiently by Pooled Patient Sera than the Wildtype

Microneutralisation assays were performed to assess the impact of the Spike mutations on neutralisation by pooled patient sera. The NTD and RBD are known to contain epitopes recognised by neutralising antibodies [15,47,48], meaning that mutations located in these regions may contribute to partial immune escape. Mutations located close to the S1/S2 site may impact cleavage efficiency, which could alter which epitopes are exposed [49]. Alternatively, these mutations may be part of antibody epitopes that are not yet understood.

**Figure 6:**
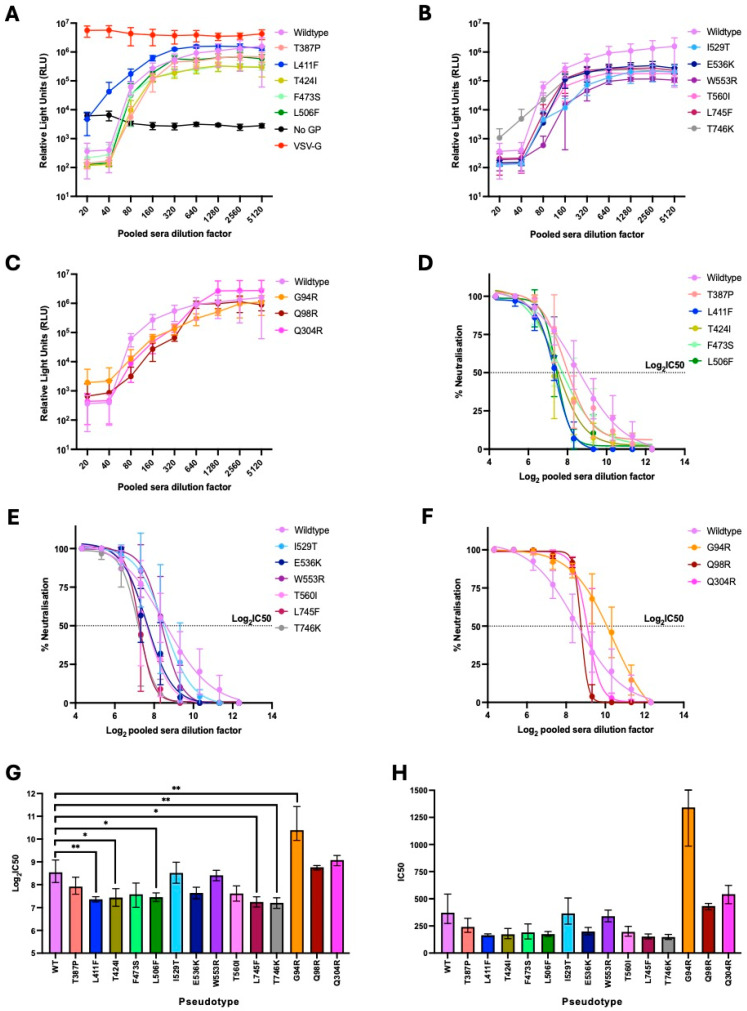
Pseudotypes carrying the L411F, T424I, L506F, L745F and T746K mutations were neutralised less efficiently than the wildtype. Pooled patient sera was serially diluted two-fold from 1:20 to 1:5120. Spike pseudotypes were diluted to 10^5^–10^6^ RLU based on titres determined previously. The MERS-CoV Spike pseudotypes, no GP and VSV-G pseudotype controls were incubated with pooled patient sera for 1 h prior to addition of HEK-293T cells expressing DPP4. (**A**–**C**) After 72 h, luciferase activity was measured using a GloMax^®^ Discover microplate reader (Promega) and raw relative light units (RLU) data were plotted against dilution factor for each pseudotype. (**D**–**F**) % Neutralisation was calculated from the raw data using the no sera control values (0% neutralisation value) and mean no GP control RLU value (100% neutralisation value). Log(inhibitor) vs. normalised response—variable slope non-linear regression curves were fitted to the % neutralisation values. (**G**) Log_2_IC_50_ values were calculated from the log(inhibitor) vs. response—variable slope curves. One-way ANOVA with Dunnet’s multiple comparisons test indicated that pseudotypes carrying the L411F, T424I, L506F, L745F and T746K mutations were neutralised less efficiently than the wildtype, while the G94R pseudotype was neutralised more efficiently than the wildtype (n = 4 independent replicates, error bars show mean values with standard deviation; * *p* < 0.05, ** *p* < 0.01). (**H**) Log_2_IC_50_ values were anti-logged to give the half maximal inhibitory dilution factor of pooled patient sera for each pseudotype.

To set up the microneutralisation assays, pooled patient sera from recovered patients that had been infected with MERS-CoV (NIBSC, [46]) was serially diluted 2-fold from 1:20 to 1:5120. Spike wildtype, Spike mutants 1–15, VSV-G and no GP pseudotypes were added at a titre of 10^5^–10^6^ RLU and incubated for 1 h with the sera. No sera controls were also included for each pseudotype. HEK-293T DPP4 cells were then added and incubated for a further 72 h. Antibodies in the pooled patient sera should bind to the Spike proteins of the pseudotypes, preventing their entry into HEK-293T DPP4 cells and resulting in reduced expression of the firefly luciferase reporter. After 72 h, luminescence was measured to determine the extent to which the pseudotypes were inhibited by the pooled patient sera (Figure 6).

Percent neutralisation values were calculated from the raw data using the no sera controls for each pseudotype as the 0% neutralisation RLU value (Figure 6A–F). The mean no GP control RLU value was set as the 100% neutralisation value. Non-linear regression curves were fitted to the percent neutralisation values using GraphPad Prism v10 software (Figure 6D–F). Log_2_ 50% inhibitory concentration (Log_2_IC_50_) values were then calculated from the non-linear regression curves (Figure 6G), as previously described [50]. Log_2_IC_50_ values were then anti-logged to give the IC_50_ values (Figure 6H). The IC_50_ values represent the pooled patient sera dilution factor that results in 50% inhibition of each pseudotype.

Statistical analysis of Log_2_IC_50_ values was performed to compare the neutralisation of mutants 1–15 with that of the wildtype Spike pseudotype. Pseudotypes carrying the L411F, T424I, L506F, L745F and T746K mutations were neutralised less efficiently by pooled patient sera than the wildtype (*p* < 0.01 T424I, T746K; *p* < 0.05 T424I, L506F, L745F) (Figure 6G). The pseudotype carrying the G94R mutation was neutralised more efficiently than the wildtype pseudotype (*p* < 0.01). Microneutralisation of the remaining Spike mutant pseudotypes was comparable to that of the wildtype. Importantly, pooled patient sera had no inhibitory effect on the VSV-G and no GP controls (Figure 6A).

## 5. Discussion

This study was conducted to investigate the phenotypic impact of Spike mutations in contemporary MERS-CoV strains to improve our understanding of human MERS-CoV infections. Fifteen Spike mutations were selected for characterisation using a contemporary MERS-CoV Spike backbone. This backbone carried five amino acid changes relative to EMC/2012: V26L, L717I, Q1020R, R1179M and E1183D (Table 1). V26L and L717L are located in the S1 subunit, which mediates host cell attachment and receptor binding [27,28,29,30,31]. Q1020R, R1179M and E1183D are located in the S2 subunit, which mediates membrane fusion [26]. The 15 SNPs characterised were located in the S1 subunit in the NTD, RBD or adjacent to the S1/S2 cleavage site. Novel naturally occurring mutations impacting fusion and neutralisation of contemporary MERS-CoV were identified in this study. The I529T, E536K and L745F mutations increased membrane fusion and syncytia formation (Figure 3 and Figure 5). Whereas the L411F, T424I, L506F, L745F and T746K mutations were shown to increase resistance to neutralisation by pooled patient sera (Figure 6).

Prior to this study, knowledge surrounding the 15 mutations was limited due to them not having been previously characterised or characterised using an EMC/2012 backbone. Kleine-Weber et al. previously demonstrated that L411F and F473S did not impact entry when introduced to the EMC/2012 Spike sequence in a VSV-based pseudotyping system [23]. Additionally, L411F was shown not to impact virus neutralisation by sera isolated from patients that had been infected with MERS-CoV in 2014 [7]. Spike residues 506, 536 and 553 have been confirmed to make direct contact with DPP4 during receptor binding [51], indicating that mutations occurring at these residues may impact entry. Tang et al. demonstrated that the L506F substitution caused a minimal reduction in DPP4 binding and was neutralised less efficiently by antibodies isolated from a human antibody phage display library [52]. However, it is important to note that the EMC/2012 sequence was used as the Spike backbone and to generate the neutralising antibodies used in this study. Residues 506, 536 and 553 were also identified as being part of the epitope recognised by the MERS-GD27 neutralising antibody isolated from a recovered patient [47]. Furthermore, when the L506F and E536K mutations were introduced to the EMC/2012 Spike sequence, the neutralisation efficiency of MERS-GD27 was significantly decreased. Previous studies have also shown that I529T, one of the mutations associated with the MERS-CoV outbreak in South Korea in 2015, was associated with a reduced affinity for the DPP4 receptor, reduced neutralisation with patient sera and reduced pathogenicity in mice [22,23,53,54,55]. Hoffman et al. demonstrated that introducing the T746K polymorphism into the EMC/2012 Spike sequence in a VSV-based pseudotyping system resulted in increased S1/S2 cleavage, without increasing entry into Calu3 cells [56]. However, when the E32A and Q1020R mutations, which are present in clade B viruses, were introduced to the EMC/2012 Spike sequence alongside T746K, entry into Calu3 cells was improved. This highlights the importance of relevant Spike backbone sequences when characterising contemporary virus strains due to mutations acting in a compensatory manner.

Luciferase-based titration assays showed that the G94R, Q98R and Q304R mutations significantly reduced pseudotype titres relative to the wildtype (Figure 2D). This could indicate impaired entry of these mutants, as these mutations are located in the NTD, which is known to be involved in host cell attachment [27,28,29]. However, it is important to note that further experiments in which pseudotypes have been normalised would be required to confirm this. Additionally, it is also possible that discrepancies in transfection efficiency and/or packaging of Spike during pseudoparticle generation may have caused the differences in titres. Split GFP cell-cell fusion assays were subsequently performed to further characterise the Spike variants.

Receptor binding and membrane fusion were enhanced for Spike proteins carrying the I529T, E536K and L745F mutations (Figure 3). This was also reflected by increased syncytia formation for these Spike variants relative to the wildtype (Figure 5). The I529T mutation was previously shown to diminish entry [53], although the reduction in entry caused by this mutation was later demonstrated to only occur in cells with low levels of DPP4 expression [23]. It is likely that the BHK-21 DPP4 cells used in this experiment expressed enough DPP4 for receptor binding and membrane fusion to occur. To add to this, a recent study by So et al. also demonstrated that the I529T mutation caused reduced entry [22], in contrast to the improved cell–cell fusion observed here. This discrepancy may be due to either the use of different cell models or to compensatory mutations present in the different Spike backbones used in each study. However, it is also important to note that syncytia formation can be independent of cell entry levels, as was observed when the Omicron variant was compared to other SARS-CoV variants in a previous study [57]. Although interestingly, So et al. identified that 2019 strains exhibited reduced entry, indicating that the 2019 backbone used here may have had a detrimental effect on entry properties, although further experiments comparing Spike backbones would be required to confirm this hypothesis [22]. Given that residue 536 makes direct contact with the DPP4 receptor [51], the improved receptor binding and membrane fusion observed for the E536K mutant in this assay may be due to improved DPP4 binding. The L745F mutation is located in close proximity to the S1/S2 cleavage site, suggesting that the improvement in receptor binding and membrane fusion observed here may be due to altered S1/S2 cleavage efficiency of this Spike variant. More efficient cleavage at S1/S2 results in more Spike proteins on the pseudoparticle surface being primed for receptor binding, which has previously been demonstrated to increase host cell entry [26].

Receptor binding and membrane fusion were reduced for Spike variants carrying the T387P, L411F, T424I, W553R, T560I and G94R mutations (Figure 3). This was also reflected by the reduced syncytia formation observed for these Spike variants relative to that of the wildtype (Figure 4 and Figure 5). Spike residues 387, 411 and 424 are not known to directly interact with the DPP4 receptor. However, these substitutions may have caused minor changes in the Spike protein structure due to changes in size and charge of the amino acid side chains, resulting in a knock-on effect on receptor binding. In contrast to the reduction in receptor binding and membrane fusion observed for the L411F variant in this study, Kleine-Weber et al. previously showed that the L411F mutation did not impact entry into HEK-293T DPP4 cells [23]. This discrepancy may be attributed to differences in the Spike backbone used in each of these studies. The L411F variant used here includes five additional Spike amino acid changes associated with clade B, lineage 5 viruses (Table 1), relative to the EMC/2012 Spike sequence that was used in the study conducted by Kleine-Weber et al.

Residues 553 and 560 are known to make direct contact with DPP4, which indicates that the reduced receptor binding and membrane fusion observed for the W553R and T560I variants may be due to reduced affinity for the receptor. In addition to reduced receptor binding/membrane fusion, the G94R variant also had a significantly lower titre than the wildtype (Figure 2 and Figure 3). Given that residue 94 is located on the outer surface of the Spike NTD (Figure 1) and that G94R is associated with a change in amino acid charge, it is possible that the G94R mutation interferes with host cell attachment, resulting in the reduced titre and syncytia formation observed for this Spike variant.

It is important to note that membrane fusion and syncytia formation may be increased or decreased independently of virus entry levels, as was demonstrated in a previous study comparing SARS-CoV-2 variants. Reduced syncytia formation was observed for Omicron, while entry level remained comparable to the other variants analysed [57]. It is also possible that the HEK-293T cells transiently expressing human DPP4 used in the cell–cell fusion assays may not accurately reflect DPP4 expression levels in the human respiratory tract. Additionally, while three independent cell–cell fusion assays were performed to account for differences in transfection efficiency, it is possible that Spike expression may have varied between mutants. For example, western blot analyses indicated a reduction in Spike expression for the T387P and L411F variants (Supplementary Figure S3), which, while not impacting the titres of these variants (Figure 2), may have impacted syncytia formation in the cell–cell fusion assays. Therefore, Spike expression would need to be quantified prior to performing cell–cell fusion assays to confirm the results observed here. Alternatively, normalisation of pseudotypes according to Spike expression prior to performing infectivity assays in a cell model that naturally expresses the DPP4 receptor, such as Calu3, would conclusively confirm the impact of these mutations on entry in contemporary MERS-CoV strains.

Pseudotypes carrying the L411F, T424I, L506F, L745F and T746K SNPs were neutralised less efficiently by pooled patient sera than the wildtype (Figure 6). In contrast to the results presented here, Schroeder et al. previously showed that the L411F SNP did not impact virus neutralisation by patient sera [7]. This difference may be due to the patient sera being obtained from patients infected with different clade B lineages. Schroeder et al. used patient sera collected in 2014, prior to the emergence of lineage 5 viruses, whereas the pooled patient sera used in this study was obtained from patients infected with lineage 5 viruses during the South Korean MERS-CoV outbreak in 2015. This may explain why the I529T mutation did not increase resistance to neutralisation in the study presented here, which does not align with some previous studies [23,54]. Although, it should also be noted that Mayer et al. recently demonstrated that sera from patients vaccinated with the MVA-MERS-S vaccine were able cross-neutralise the I529T variant, indicating no neutralisation escape of this variant [58]. The I529T mutation arose during the MERS-CoV outbreak in South Korea and, prior to the study by Mayer et al., was associated with reduced neutralisation relative to sera raised against EMC/2012 and early clade B viruses. However, the pooled patient sera used in the study presented here was obtained from patients infected with lineage 5 strains that were likely carrying the I529T substitution, hence increased resistance to neutralisation was not observed.

The T424I pseudotype was neutralised less efficiently by pooled patient sera than the wildtype (Figure 6). This residue has not previously been identified as being located within an antibody epitope, although its location on the outer surface of the RBD (Figure 1) indicates that it could be targeted by neutralising antibodies. The L506F and E536K mutations have previously been shown to reduce neutralisation by sera obtained from patients infected with MERS-CoV during the South Korean outbreak in 2015 [47]. These residues have been confirmed to be located within neutralising antibody epitopes and the E536K mutation was found to reduce neutralisation to a lesser extent than the L506F mutation [47,52]. In this study, the L506F mutation significantly reduced neutralisation by pooled patient sera (Figure 6), restoring the ability of this mutant to bind DPP4 and infect host cells. The E536K mutation also appeared to reduce neutralisation, although this was found not to be statistically significant in this study (Figure 6).

The L745F and T746K pseudotype variants were neutralised less efficiently by pooled patient sera than the wildtype (Figure 6). Residues 745 and 746 are located within close proximity to the S1/S2 cleavage site (Figure 1), and S1/S2 cleavage can occur during biosynthesis of the Spike protein or upon interaction with target host cells [25,26]. Differences in cleavage efficiency will impact the proportion of primed versus unprimed Spike proteins in pseudotype variants, which may alter the epitopes exposed to neutralising antibodies [49]. Interestingly, the L745F mutation was also found to increase receptor binding and membrane fusion in this study, which means that this substitution appears to have made MERS-CoV more infectious and provided increased resistance to neutralisation (Figure 3 and Figure 6).

While novel mutations impacting neutralisation have been identified here, it is important to note that the pooled patient sera used in this study was obtained from two individuals who were infected with MERS-CoV in 2015. Further analyses incorporating patient sera collected in distinct locations and across different years would be required to obtain a more thorough understanding of Spike mutations that increase resistance to neutralisation in the wider population.

To summarise, a contemporary MERS-CoV pseudotyping system was developed and the phenotypic consequences of naturally occurring Spike mutations on receptor binding, membrane fusion and neutralisation were identified. This pseudotyping system can continue to be used to characterise Spike mutations as they arise or could be used in the development of medical countermeasures for MERS-CoV. Continued investigation of genotype-to-phenotype consequences in MERS-CoV is essential for pandemic preparedness and reducing the risk to public health.

## Figures and Tables

**Table 1:** Summary of Spike amino acid changes present in the 2019 clinical isolate relative to the EMC/2012 reference strain. An alignment of the Spike sequences carried by EMC/2012 and the 2019 clinical isolate was performed and amino acid differences are listed in the table below.

Spike Amino Acid No.	EMC/2012 Amino Acid	Clinical Isolate 2019 Amino Acid
387	P	T
717	L	I
1020	Q	R
1179	R	M
1183	E	D

**Table 2:** Summary of Spike mutations of interest that were selected for characterisation. Pseudotypes were assigned a mutant number and the location of each mutation is listed in the table below. The wildtype carried a clade B lineage 5 Spike sequence, which had five amino acid changes relative to the EMC/2012 reference strain. Mutants were generated via site-directed mutagenesis of the wildtype Spike plasmid.

Pseudotype	Spike Mutation	Location of Mutation
Wildtype	Clinical sample (Saudi Arabia, 2019)	5 Spike aa changes vs. EMC/2012 strain
Mutant 1 T387P	T387P	RBD
Mutant 2 L411F	L411F	RBD
Mutant 3 T424I	T424I	RBD
Mutant 4 F473S	F473S	RBD
Mutant 5 L506F	L506F	RBD
Mutant 6 D510G	D510G	RBD
Mutant 7 I529T	I529T	RBD
Mutant 8 E536K	E536K	RBD
Mutant 9 W553R	W553R	RBD
Mutant 10 T560I	T560I	RBD
Mutant 11 L745F	L745F	Adjacent to S1/S2
Mutant 12 T746K	T746K	Adjacent to S1/S2
Mutant 13 G94R	G94R	NTD
Mutant 14 Q98R	Q98R	NTD
Mutant 15 Q304R	Q304R	NTD

## Data Availability

The original contributions presented in this study are included in the article/Supplementary Material. Further inquiries can be directed to the corresponding authors.

## Supplementary Materials

The following supporting information can be downloaded at: https://www.mdpi.com/article/10.3390/v18030377/s1, Table S1: Spike mutagenesis primer sequences; Table S2: Primary and secondary antibodies used for Western blot analysis; Figure S1: Wildtype Spike sequence of representative MERS-CoV clade B virus isolated from a patient in Saudi Arabia in 2019; Figure S2: Western blot analysis of wildtype Spike pseudotype cell lysate confirmed expression of Spike protein, HIV-1 gag/pol lentiviral core proteins and the firefly luciferase reporter; Figure S3: Western blot analysis of Spike pseudotype mutant 1–12 cell lysates confirmed expression of Spike protein, HIV-1 gag/pol lentiviral core proteins and the firefly luciferase reporter; Figure S4: Western blot analysis of Spike pseudotype mutant 13–15 cell lysates confirmed expression of Spike protein, HIV-1 gag/pol lentiviral core proteins and the firefly luciferase reporter.
