# Supplementary material for: Characterisation of Naturally Occurring MERS-CoV Spike Mutations and Their Impact on Fusion and Neutralisation
Source: Viruses. 2026 Mar 18;18(3):377. doi: 10.3390/v18030377 (PMC13030573; doi:10.3390/v18030377)
Supplement: Supplementary file 1 [file viruses-18-00377-s001.zip › viruses-4190704-supplementary.pdf]

Supplementary Methods

Western blot

Proteins were transferred onto a polyvinylidene difluoride (PVDF) membrane using Trans-Blot® Turbo™ mini PVDF transfer packs (BioRad) and the Trans-Blot® Turbo™ transfer system (BioRad) using the manufacturer's settings for 1 Mini-PROTEAN® TGX gel per tray. Membranes were then blocked in 5% non-fat milk powder dissolved in tris-buffered saline with tween-20 (TBS-T; 20mM Tris, 150mM of NaCl, 0.1% Tween 20, pH 7.6) for 1h at room temperature. Membranes were incubated with appropriate primary antibodies diluted in blocking buffer at 4°C overnight (Supplementary Table 2). Prior to incubation with secondary antibodies, membranes were washed three times with TBS-T for 5 minutes per wash. Appropriate horseradish peroxidase conjugated secondary antibodies were diluted in blocking buffer and incubated with membranes for 1h at room temperature (Supplementary Table 2). Membranes were washed five times with TBS-T for 5 minutes per wash prior to the addition of enhanced chemiluminescent (ECL) substrate (BioRad) and imaging with a ChemiDoc imaging system (BioRad). After initial probing, membranes were stripped at room temperature as follows: two 10 minute incubations with stripping buffer (15g Glycine, 1g SDS, 10mL Tween 20, made up to 1L with double-distilled water d(dH_20); pH 2.2), two 10 minute washes in PBS (Sigma-Aldrich) and two five minute washes in TBS-T. Membranes were then blocked and probed for the GAPDH loading control as described above.

Supplementary Tables and Figures

Supplementary Table S1: Spike mutagenesis primer sequences. *Mutations were introduced to the wildtype spike plasmid via site-directed mutagenesis using the primers listed in the table.*

Pseudotype	Primer	Primer Sequence 5' – 3'
Mutant 1<br>T387P	T387P_F<br>T387P_R	AATGCGACTTCAGCCCCCTGCTGTCTGGC<br>GCCAGACAGCAGGGGGCTGAAGTCGCATT
Mutant 2<br>L411F	L411F_F<br>L411F_R	GTTCACCAACTGCAATTACAACTTTACCAAGCTGCTGAGCC<br>GGCTCAGCAGCTTGGTAAAGTTGTAATTGCAGTTGGTGAAC
Mutant 3<br>T424I	T424I_F<br>T424I_R	TCCGTGAACGACTTCATCTGTAGCCAGATCAGC<br>GCTGATCTGGCTACAGATGAAGTCGTTCACGGA
Mutant 4<br>F473S	F473S_F<br>F473S_R	AACTACAAGCAGTCCTCCAGCAACCCTACCTGC<br>GCAGGTAGGGTTGCTGGAGGACTGCTTGTAGTT
Mutant 5<br>L506F	L506F_F<br>L506F_R	ATCAACAAGTGCAGCAGATTTCTGAGCGACGACAGAACC<br>GGTTCTGTCGTCGCTCAGAAATCTGCTGCACTTGTTGAT
Mutant 6<br>D510G	D510G_F<br>D510G_R	ACTGCTGAGCGACGGCAGAACCGAAGTGC<br>GCACTTCGGTTCTGCCGTCGCTCAGCAGT
Mutant 7<br>I529T	I529T_F<br>I529T_R	CCCTTGCGTGTCCACCGTGCCTAGCAC<br>GTGCTAGGCACGGTGGACACGCAAGGG
Mutant 8<br>E536K	E536K_F<br>E536K_R	CTAGCACCGTTTGGAAGGACGGCGACTAC<br>GTAGTCGCCGTCCTTCCAAACGGTGCTAG
Mutant 9<br>W553R	W553R_F<br>W553R_R	GGAAGGCGGAGGACGGCTGGTGGC<br>GCCACCAGCCGTCCTCCGCCTTCC
Mutant 10<br>T560I	T560I_F<br>T560I_R	GCTGGTGGCCTCTGGATCTATAGTGGCCATGA<br>TCATGGCCACTATAGATCCAGAGGCCACCAGC
Mutant 11<br>L745F	L745F_F<br>L745F_R	GCTCTGCCAGATACACCCATCACATTTACCCCAAGATCCG<br>CGGATCTTGGGGTAAATGTGATGGGTGTATCTGGCAGAGC
Mutant 12<br>T746K	T746K_F<br>T746K_R	GCCAGATACACCCATCACACTGAAACCAAGATCCGTG<br>CACGGATCTTGGTTTCAGTGTGATGGGTGTATCTGGC
Mutant 13<br>G94R	G94R_F<br>G94R_R	CGGACACGCCACCCGGACCACACCTCAGA<br>TCTGAGGTGTGGTCCGGGTGGCGTGTCCG
Mutant 14<br>Q98R	Q98R_F<br>Q98R_R	CGGCACCACACCTCGGAAACTGTTCGTGG<br>CCACGAACAGTTTCCGAGGTGTGGTGCCG
Mutant 15<br>Q304R	Q304R_F<br>Q304R_R	GCATCCGGTCCATCCGGAGCGACAGAAAAGC<br>GCTTTTCTGTCGCTCCGGATGGACCGGATGC

Supplementary Table S2: Primary and secondary (*) antibodies used for Western blot analysis. *Antibodies targeting the different pseudotype proteins were used at the dilutions listed in the table to confirm expression.*

**Target**	**Vendor**	**Cat. number**	**Dilution**
MERS-CoV Spike S1	Thermo Fisher	PA5-119581	1/10000
Firefly luciferase	Abcam	Ab185923	1/2000
HIV-1 p24	Abcam	Ab32352	1/2000
VSV-G	GenScript	A00199	1/4000
GAPDH	Abcam	Ab8245	1/5000
Goat anti-rabbit*	Sigma	A6154	1/5000
Goat anti-mouse*	Sigma	A4416	1/5000

Supplementary Figure S1: Wildtype Spike sequence of representative MERS-CoV clade B virus isolated from a patient in Saudi Arabia in 2019.

ATGATACACTCAGTGTTTCTACTGATGTTCTTGTTAACACCTACAGAAAGTTACGTTGAT
GTAGGGCCAGATTCTGTTAAGTCTGCTTGTATTGAGGTTGATATACAACAGACTTTCTTT
GATAAAACTTGGCCTAGGCCAATTGATGTTTCTAAGGCTGACGGTATTATATACCCTCAA
GGCCGTACATATTCTAACATAACTATCACTTATCAAGGTCTTTTTCCCTATCAGGGAGAC
CATGGTGATATGTATGTCTACTCTGCAGGACATGCTACAGGCACAACTCCACAAAAGTT
GTTTGTAGCTAACTATTCTCAGGACGTCAAACAGTTTGCTAATGGGTTTGTCGTCCGTAT
AGGAGCAGCTGCCAATTCCACTGGCACTGTTATTATTAGCCCATCTACCAGCGCTACTA
TACGAAAAATTTACCCTGCTTTTATGCTGGGTTCTTCAGTTGGTAATTTCTCAGATGGTA
AAATGGGCCGCTTCTTCAATCATACTCTAGTTCTTTTGCCCGATGGATGTGGCACTTTAC
TTAGAGCTTTTTATTGTATTCTAGAGCCTCGCTCTGGAAATCATTGTCCTGCTGGCAATT
CCTATACTTCTTTTGCCACTTATCACACTCCTGCAACAGATTGTTCTGATGGCAATTACA
ATCGTAATGCCAGTCTGAACTCTTTTAAGGAGTATTTTAATTTACGTAACTGCACCTTTAT
GTACACTTATAACATTACCGAAGATGAGATTTTAGAGTGGTTTGGCATTACACAAACTGC
TCAAGGTGTTCACCTCTTCTCATCTCGGTATGTTGATTTGTACGGCGGCAATATGTTTCA
ATTTGCCACCTTGCCTGTTTATGATACTATTAAGTATTATTCTATCATTCCTCACAGTATT
CGTTCTATCCAAAGTGATAGAAAAGCTTGGGCTGCCTTCTACGTATATAAACTTCAACC
GTTAACTTTCCTGTTGGATTTTTCTGTTGATGGTTATATACGCAGAGCTATAGACTGTGG
TTTTAATGATTTGTCACAACTCCACTGCTCATATGAATCCTTCGATGTTGAATCTGGAGT
TTATTCAGTTTCGTCTTTCGAAGCAAAACCTTCTGGCTCAGTTGTGGAACAGGCTGAAG
GTGTTGAATGTGATTTTTCAACTCTTCTGTCTGGCACACCTCCTCAGGTTTATAATTTCA
AGCGTTTGGTTTTTACCAATTGCAATTATAATCTTACCAAATTGCTTTCACTTTTTTCTGT
GAATGATTTTACTTGTAGTCAAATATCCCCAGCAGCAATTGCTAGCAACTGTTATTCTTC
ACTGATTTTGGATTATTTTTCATACCCACTTAGTATGAAATCCGATCTCAGTGTTAGTTCT
GCTGGTCCAATATCCCAGTTTAATTATAAACAGTCTTTTTCTAATCCCACTTGTTTGATTT
TAGCGACTGTTCCTCATAACCTTACTACTATTACTAAGCCTCTTAAGTACAGCTATATTAA
CAAGTGCTCTCGTCTTCTTTCTGATGATCGTACTGAAGTACCTCAGTTAGTGAACGCTAA
TCAATACTCACCCTGTGTATCCATTGTCCCATCCACTGTGTGGGAAGACGGTGATTATT
ATAGGAAACAACTATCTCCACTTGAAGGTGGTGGCTGGCTTGTTGCTAGTGGCTCAACT
GTTGCCATGACTGAGCAATTACAGATGGGCTTTGGTATTACAGTTCAATATGGTACAGA
CACCAATAGTGTTTGCCCCAAGCTTGAATTTGCTAATGACACAAAAATTGCCTCTCAATT
AGGCAATTGCGTGGAATATTCCCTCTATGGTGTTTCGGGCCGTGGTGTTTTTCAGAATT
GCACAGCTGTAGGTGTTCGACAGCAGCGCTTTGTTTATGATGCGTACCAGAATTTAGTT
GGCTATTATTCTGATGATGGCAACTACTACTGTTTGCGTGCTTGTGTTAGTGTTCCTGTT
TCTGTCATCTATGATAAAGAAACTAAAACCCACGCTACTCTATTTGGTAGTGTTGCATGT

GAACACATTTCCTCTACCATGTCTCAATACTCCCGTTCTACGCGATCAATGCTTAAACGG
CGAGATTCTACATATGGTCCCCTTCAGACACCTGTTGGTTGTGTCCTAGGAATTGTTAAT
TCCTCTTTGTTCGTAGAGGACTGCAAGTTGCCTCTTGGTCAATCTCTCTGTGCTCTTCCT
GACACACCTAGTACTCTCACACCTCGCAGTGTGCGCTCTGTTCCAGGTGAAATGCGCTT
GGCATCCATTGCTTTTAATCATCCTATTCAGGTTGATCAACTTAATAGTAGTTATTTTAAA
TTAAGTATACCTACTAATTTTTCCTTTGGTGTGACTCAGGAGTACATTCAGACAACCATT
CAGAAAGTTACTGTTGATTGTAAACAGTACGTTTGCAATGGTTTCCAGAAGTGTGAGCA
ATTACTGCGCGAGTATGGCCAGTTTTGTTCCAAAATAAACCAGGCTCTCCATGGTGCCA
ATTTACGCCAGGATGATTCTGTACGTAATTTGTTTGCGAGCGTGAAAAGCTCTCAATCAT
CTCCTATCATACCAGGTTTTGGAGGTGACTTTAATTTGACACTTCTAGAACCTGTTTCTA
TATCTACTGGCAGTCGTAGTGCACGTAGTGCTATTGAGGATTTGCTATTTGACAAAGTC
ACTATAGCTGATCCTGGTTATATGCAAGGTTACGATGATTGTATGCAGCAAGGTCCAGC
ATCAGCTCGTGATCTTATTTGTGCTCAATATGTGGCTGGTTATAAAGTATTACCTCCTCT
TATGGATGTTAATATGGAAGCCGCGTACACTTCATCTTTGCTTGGCAGCATAGCAGGTG
TTGGCTGGACTGCTGGCTTATCCTCCTTTGCTGCTATTCCATTTGCACAGAGTATTTTTT
ATAGGTTAAACGGTGTTGGCATTACTCAACAGGTTCTTTCAGAGAACCAAAAGCTTATTG
CCAATAAGTTTAATCAGGCTCTGGGAGCTATGCAAACAGGCTTCACTACAACTAATGAA
GCTTTTCGGAAGGTTCAGGATGCTGTGAACAACAATGCACAGGCTCTATCCAAATTAGC
TAGCGAGCTATCTAATACTTTTGGTGCTATTTCCGCCTCTATTGGAGACATCATACAACG
TCTTGATGTTCTCGAACAGGACGCCCAAATAGACAGACTTATTAATGGCCGTTTGACAA
CACTAAATGCTTTTGTTGCACAGCAGCTTGTTCGTTCCGAATCAGCTGCTCTTTCCGCT
CAATTGGCTAAAGATAAAGTCAATGAGTGTGTCAAGGCACAATCCAAGCGTTCTGGATT
TTGCGGTCAAGGCACACATATAGTGTCCTTTGTTGTAAATGCCCCTAATGGCCTTTATTT
TATGCATGTTGGTTATTACCCTAGCAACCACATTGAGGTTGTTTCTGCTTATGGTCTTTG
CGATGCAGCTAACCCTACTAATTGTATAGCCCCTGTTAATGGCTACTTTATTAAAACTAA
TAACACTATGATTGTTGATGATTGGTCATATACTGGCTCGTCCTTCTATGCACCTGAGCC
CATCACCTCTCTTAATACTAAGTATGTTGCACCACAGGTGACATACCAAAACATTTCTAC
TAACCTCCCTCCTCCTCTTCTCGGCAATTCCACCGGGATTGACTTCCAAGATGAGTTGG
ATGAGTTTTTCAAAAATGTTAGCACCAGTATACCTAATTTTGGTTCTCTAACACAGATTAA
TACTACATTACTCGATCTTACCTACGAGATGTTGTCTCTTCAACAAGTTGTTAAAGCCCT
TAATGAGTCTTATATAGACCTTAAAGAGCTTGGCAATTATACTTATTACAACAAATGGCC
GTGGTACATTTGGCTTGGTTTCATTGCTGGGCTTGTTGCCTTAGCTCTATGCGTCTTCTT
CATACTGTGCTGCACTGGTTGTGGCACAAACTGTATGGGAAAACTTAAGTGTAATCGTT
GTTGTGATAGATACGAGGAATACGACCTCGAGCCGCATAAGGTTCATGTTCACTAA

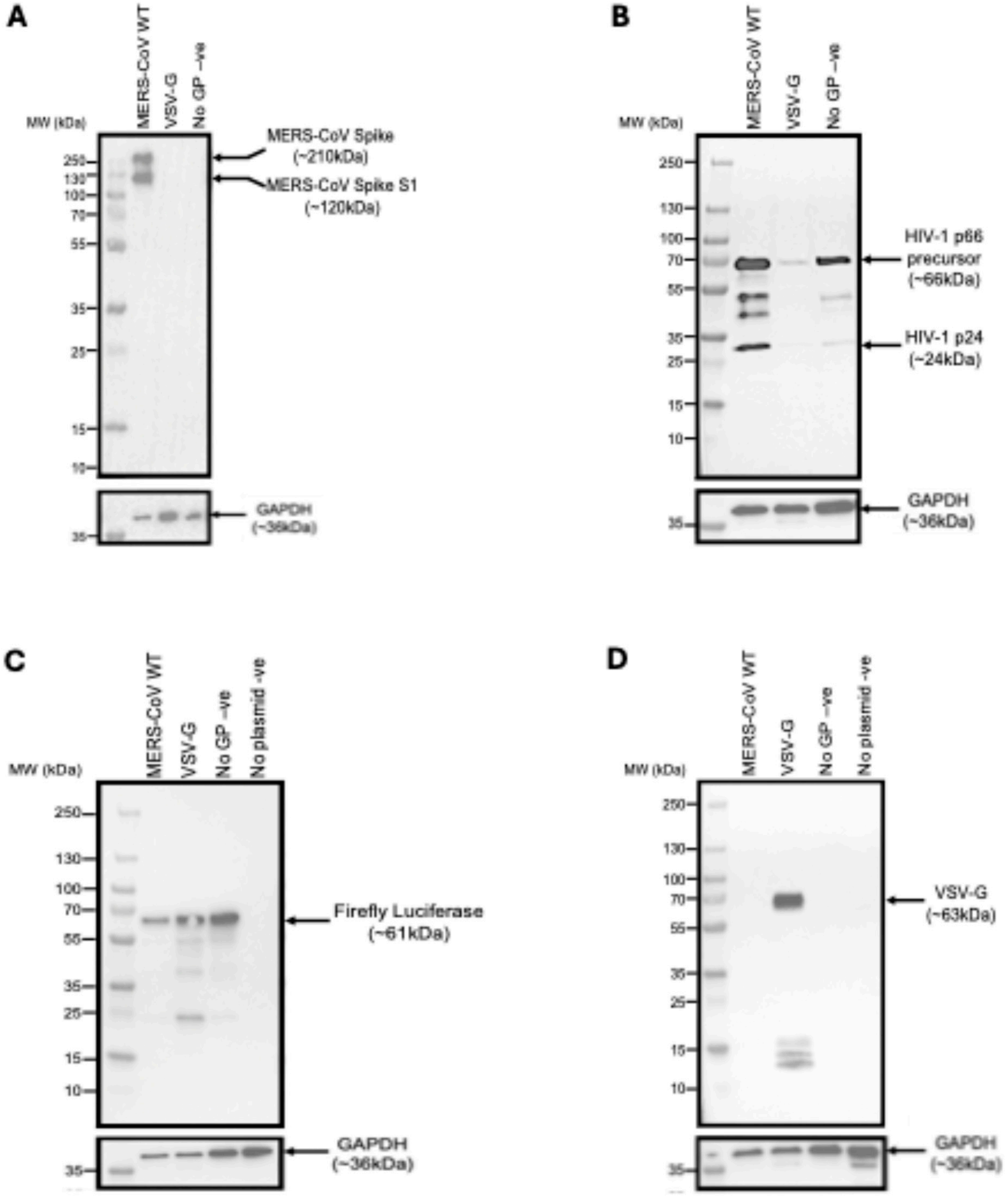

Supplementary Figure S2: Western blot analysis of wildtype Spike pseudotype cell lysate confirmed expression of Spike protein, HIV-1 gag/pol lentiviral core proteins and the firefly luciferase reporter. *Cell lysates were harvested after generation of wildtype Spike, VSV-G positive control and no GP negative control pseudotypes. Cell lysates from a no plasmid transfection control were also harvested. Cell lysates were used for Western blot analysis to confirm expression of (A) Spike, (B) HIV-1 gag/pol lentiviral core, (C) firefly luciferase reporter and (D) VSV-G. GAPDH was also included as a loading control (A-D).*

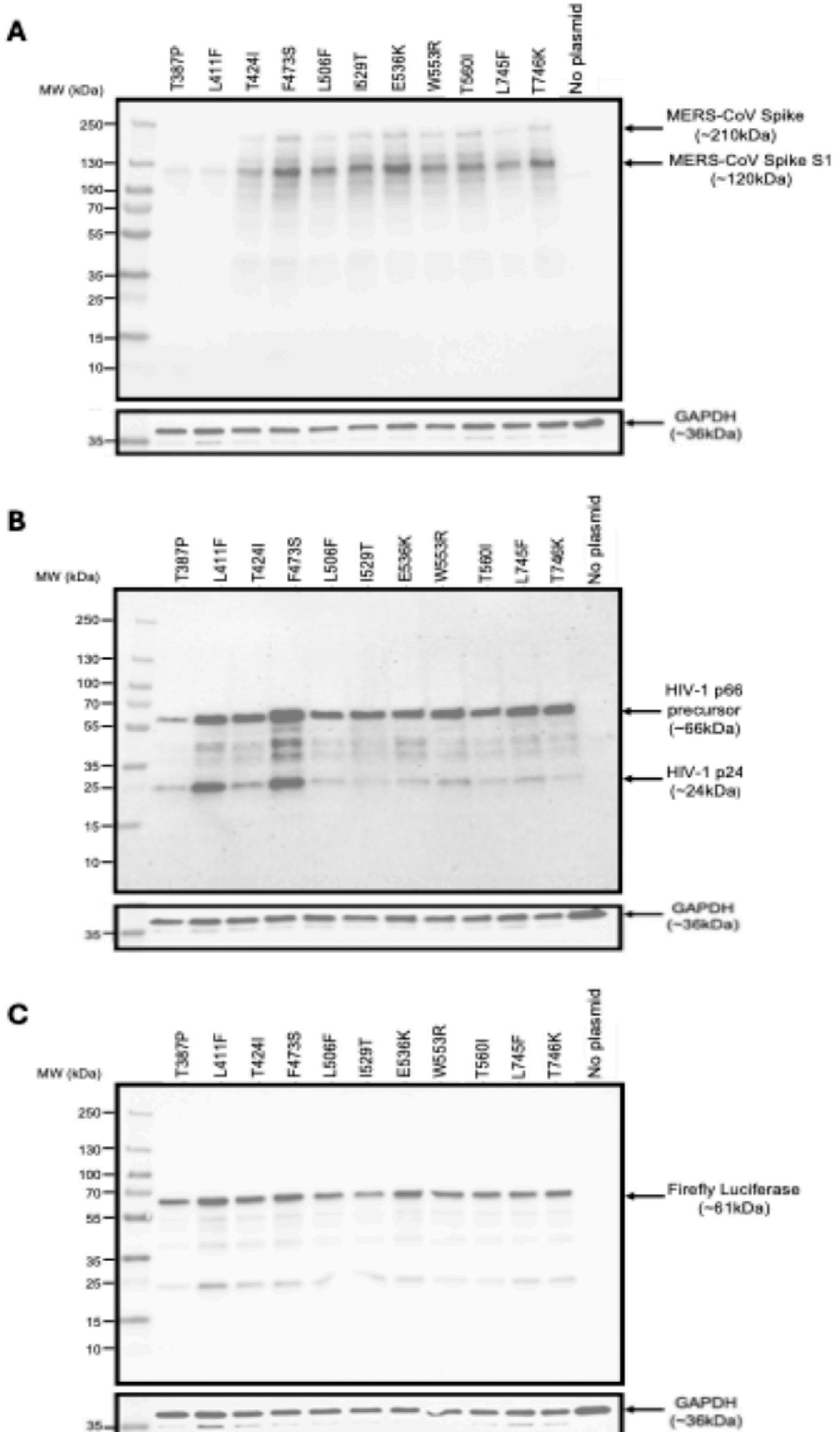

Supplementary Figure S3: Western blot analysis of Spike pseudotype mutant 1 – 12 cell lysates confirmed expression of Spike protein, HIV-1 gag/pol lentiviral core proteins

and the firefly luciferase reporter. *Cell lysates were harvested after generation of Spike pseudotype mutants 1 – 12. Cell lysates from a no plasmid transfection control were also harvested to be included as a negative control. Cell lysates were used for Western blot analysis to confirm expression of (A) Spike, (B) HIV-1 gag/pol lentiviral core and (C) the firefly luciferase reporter. (A-D) GAPDH was included as a loading control.*

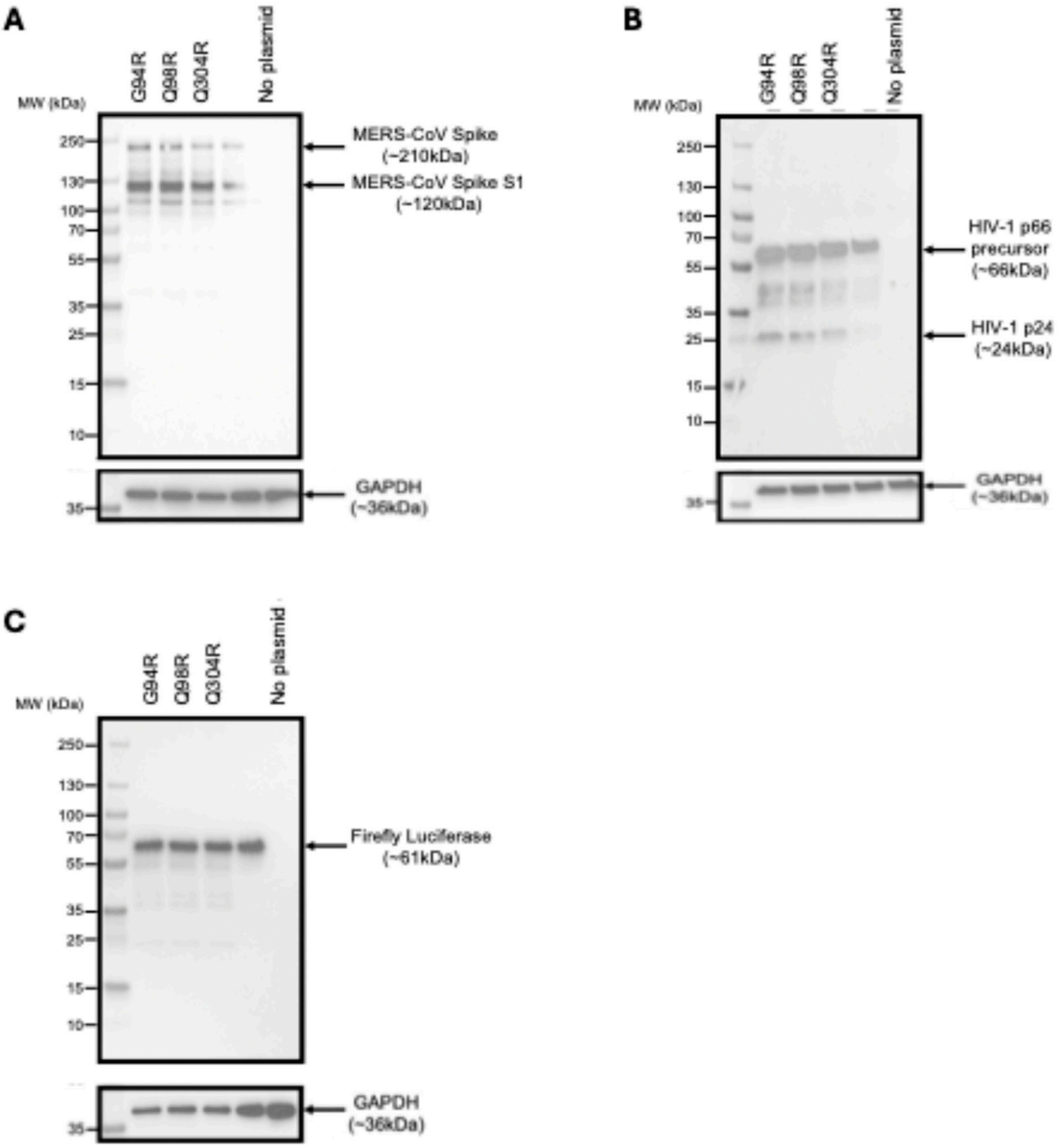

Supplementary Figure S4: Western blot analysis of Spike pseudotype mutant 13 – 15 cell lysates confirmed expression of Spike protein, HIV-1 gag/pol lentiviral core proteins and the firefly luciferase reporter. *Cell lysates were harvested after generation of*

Spike pseudotype mutants 13 – 15. Cell lysates from a no plasmid transfection control were also harvested to be included as a negative control. Cell lysates were used in Western blot analysis to confirm expression of (A) Spike, (B) HIV-1 gag/pol lentiviral core and (C) the firefly luciferase reporter. GAPDH was also included as a loading control (A-D). The unlabelled samples on these blots are not relevant to this study.
